# Vitamin D supplementation and cardiac tissue inflammation in obese rats

**DOI:** 10.1186/s40795-022-00652-2

**Published:** 2022-12-27

**Authors:** Farnoosh Ebrahimzadeh, Mahdieh Abbasalizad Farhangi, Ayda Zahiri Tausi, Mahsa Mahmoudinezhad, Mehran Mesgari-Abbasi, Faria Jafarzadeh

**Affiliations:** 1 Department of Internal Medicine, Faculty of Medicine, Mashhad University of Medical Sciences, Mashahd, Iran; 2 Department of Community Nutrition, Faculty of Nutrition, Tabriz University of Medical Sciences, Attar Neyshabouri Street, Tabriz, Iran; 3 Razavi Research Center, Razavi Hospital, Imam Reza International University, Mashahd, Iran; 4 Student Research Committee, Tabriz University of Medical Sciences, Tabriz, Iran; 5 Drug Applied Research Center, Tabriz University of Medical Sciences, Tabriz, Iran; 6 Department of Internal Medicine, School of Medicine, North Khorasan University of Medical Sciences, Bojnourd, Iran

**Keywords:** TGF- β, MCP-1, NF-κB, Obesity, Cardiac tissue

## Abstract

**Objective:**

The current study was aimed to evaluate the effects of active form of vitamin D on TGF- β, NF-κB and MCP-1 in heart tissue of obese rats.

**Methods:**

Forty rats were allocated into groups of normal diet and high fat diet for sixteen weeks; then each group was divided into two groups that received either 500 IU/kg vitamin D or placebo for five weeks. Biochemical parameters were assessed by ELISA kits.

**Results:**

Vitamin D reduced TGF-β in obese rats supplemented with vitamin D compared with other groups (*P* = 0.03). Moreover, vitamin D reduced MCP-1 concentrations in the heart tissues of both vitamin D administered groups compared to placebo one (*P* = 0.002). NF-κB in the heart of HFD + vitamin D group was significantly lower (*P* = 0.03). Current study also showed that vitamin D improves glycemic status and reduce insulin resistance significantly in HFD group (*P* = 0.008).

**Conclusion:**

Vitamin D was a potential anti- inflammatory mediator of cardiovascular disease and markers of glycemic status in obese rats. Further investigations are needed to better identify the therapeutic role of this vitamin in CVD and to elucidate the underlying mechanisms.

## Introduction

Overweight and obesity lead to adverse metabolic consequences like hypertension, hypercholesterolemia, and hypertriglyceridemia and insulin resistance [1, 2]. In fact, the mortality and morbidity of cardiovascular disease (CVD) is meaningfully higher among obese individuals. The possible reason is that the enlarged adipose tissue mass in obese individuals is a potent source of numerous peptides or nonpeptides that play a role in cardiovascular homeostasis; these molecules including interleukin (IL)-6, tumor necrosis factor (TNF)-α, transforming growth factor (TGF)-β, monocyte chemoattractant protein (MCP)-1 and nuklear factor kappa (NF-κB) are secreted from adipose tissue and have inflammatory actions against cardiac health [3]. It is clear that several inflammatory mediators have a critical role in the pathogenesis of obesity-related cardiovascular events and blockage in their expression is considered as a target of CVD treatment. TGF-β is expressed at high concentrations in the heart tissue of fetus and adults. In the rat heart, TGF-β is located in cardio-myocytes and extracellular matrix [4]. It is involved in the cardiac valve morphogenesis and is a potent activator of fibroblasts [5, 6]. TGF-β involves in myocardial injury [7] and contributes in unresolved cardiac pro-fibrotic remodeling in heart failure [8–10]. Moroever, TGF-β and MCP-1 act in the pathogenesis of cardiac events and myocardial infarction both together; MCP-1 stimulates TGF- β production in infracted heart. Moreover, MCP-1 induces TGF- β secretion, stimulates collagen synthesis and increases fibrogenic potential of mature macrophages [11]. MCP-1, attracts mononuclear cells and is involved in the atherosclerosis [12]. Blocking the MCP-1 expression and secretion reduces the severity of myocardial inflammation in myocardial events [13].

NFκB, another inflammatory protein involved in the expression and production of MCP-1, is a potent stimulator of inflammatory process in cardiovascular disease; its activation stimulates the transcription of several inflammatory genes such as MCP-1, TNF-α, TGF-β and IL-6 and promotes plaque formation [14]. NFκB in cardiac myocytes is activated in response to IL-1β, hydrogen peroxide and myocardial ischemia and promotes cardiac hypertrophy and nitric oxide production and cardiovascular events [15–17]. Above mentioned explanation highlighted the role of integrated inflammatory molecules including MCP-1, NF-κB and TGF-β in the pathogenesis of CVD; therefore, inhibition of their production and blocking their expression could be a therapeutic approach in treatmet of CVD.

Beside the classic role of vitamin D in the growth and mineralization of bone, this steroid endocrine hormone has other health benefits [18, 19]. Several evidence show that vitamin D deficiency can be associated with cardiovascular morbidity and mortality [20, 21]. It has been shown that, 25-OHD deficiency and parathyroid hormone (PTH) excess are associated with risk of cardiovascular diseases through divergent pathways among older adults [22, 23]. Similarly, Valcour et al. pointed to a smoothly decreasing relationship between vitamin D and PTH level; increasing serum 25-OHD levels are associated with decreasing PTH levels [24].

A follow-up study revealed higher death rate among vitamin D –deficient individuals [25]. Vitamin D receptor (VDR) is expressed in cardiovascular system [26] and clinical evidence have reported that vitamin D exerts a beneficial role in cardiac remodeling and heart failure survival [27–29]. Vitamin D inhibits TGF-β -induced cardiac tissue atrophy [10] and pro-fibrotic effects of TGF-β [30] and reduces TGF-β and MCP-1 expression in vivo [31].

Although there are some inconsistent results about the effects of vitamin D supplementation on cardiovascular survival; however, a meta-analysis in this regard elucidated that vitamin D supplementation inhibits ventricular remodelling and improves cardiac function in patients with heart failure [32]. Similarly, C D’Amore’s et al. review reported that vitamin D deficiency may favor the onset and/or progression of heart failure and left ventricular remodeling and is associated with more adverse prognosis in heart failure patients [33]. Also, in the study of Gostman et al. vitamin D deficiency was highly prevalent in heart failure patients and was a significant predictor of reduced survival. While, vitamin D supplementation was associated with improved outcome [34]. As far as we know, the pivotal role of vitamin D in CVD is not understood well. In the previous published work of the current project [35], we observed the significant potential effects of vitamin D on reducing TNF-α and oxidative stress. In the current study, we aimed to evaluate the role of vitamin D on glycemic status and cardiac tissue concentrations of TGF-β, MCP-1 and NF-κB in obese rats.

## Methods

### Animals

We purchased forty rats with 200–220 g of weight from the Pasteur institute animal care center (Karaj, Iran). In the standard condition of temperature and ad libitum food, animals were housed. The study procedure was in accordance of National Institutes of Health ethical guidelines. The ethics committee registration code is TBZMED.REC.1400.068. The study is also reported in accordance with ARRIVE guidelines. Rats were fed a standard diet for one week, then were randomly assigned into two groups of normal diet (ND) or high fat diet (HFD) for four months [36]. Then after 4 months, both groups were randomly allocated in to subgroups of ND, ND + vitamin D, HFD and HFD + vitamin D [e.g. 500 IU/kg/d vitamin D or Migliol as placebo (Sigma Adrich, USA)] for five weeks.

Rats’ anesthesia procedure was performed after one week acclimation period to prevent stress-induced response. After 24 h fasting, rats were anesthetized with peritoneal injection of 6.6 mg/kg Ketamin and 0.3 mg/ kg of Xylazine. Cardiac puncture blood samples were obtained and centrifuged for sera extraction. Consequently, the rats were sacrificed by decapitation under guillotine consisting of a metal frame and a sharp blade, and being operated by one hand. After decapitation of animals, the hemisphere of heart samples were removed for further assays.

### ELISA

After homogenization of heart tissues in the phosphate-buffered saline and centrifuging them, the clear supernatants were collected for evaluation of total protein, MCP-1, NF-κB and TGF-β concentrations (e.g. Pars Azmun, Karaj and Hangzhou Eastbiopharm, China). Fasting serum glucose were assessed by UV-VIS spectrophotometer (Ultra-spec 2000, Pfizer, USA). Vitamin D and insulin was determined by ELISA kits (Eastbiopharm, China). Homeostatic model assessment of insulin resistance (HOMA-IR) was calculated as below:

$$HOMA-IR=\left[\mathrm{Fasting}\;\mathrm{serum}\;\mathrm{insulin}\;(\mu\mathrm u/\mathrm{ml})\;\times\;\mathrm{Fasting}\;\mathrm{serum}\;\mathrm{glucose}\;(\mathrm{mmol}\;/\mathrm{lit})\right]/22.5$$

### Statistical assay

SPSS software version 16 was used for statistical assays. Kolmogorov–Smirnov test was used to check the normality of variables. Data were presented as the mean ± SD. One-way analysis of variance (ANOVA) followed by *the post hoc* Tukey’s tests was used to compare the values between multiple groups. Paired sample *t* test was used to compare the parameters before and after supplementation. *P* values < 0.05 were considered as significance level.

## Results

The mean value of measured parameters are presented in Table 1 in all study groups. *Post hoc* analysis illustrated a significant difference in terms of TGF-β in the heart tissues of vitamin D-administered obese rats between HFD + vitamin D and HFD and ND + vitamin D groups (*P* = 0.03). Also, intergroup comparisons showed a significant difference between HFD and HFD + vitamin D groups (*P* = 0.002) and between ND and ND + vitamin D groups (*P* = 0.03). Accordingly, cardiac tissue concentrations of NF-κB in the vitamin D-administered obese rats was lower compared with HFD group (*P* = 0.03). Also, the NF-κB concentrations in HFD group was significantly higher than ND group (*P* = 0.04). The correlation matrix between inflammatory parameters in the cardiac tissue and markers of glycemic status (Table 2) showed a strong and significant association between cardiac TGF-β and insulin concentrations (*r* = 0.63, *P* = 0.04). No significant changes in fasting serum glucose in HFD compared to ND group was observed while there was an increase in serum insulin concentrations (*P* = 0.04) and HOMA-IR index (*P* = 0006) in HFD induced obese rats compared to ND group. Moreover, vitamin D-administered group of obese rats revealed reduced insulin (*P* = 0.01) and HOMA-IR values (*P* = 0.008) compared to HFD group. However, no significant changes was shown for glucose level after vitamin D administration (*P* > 0.05) (Table 1).

**Table 1:** Changes in weight, TGF-β, MCP-1, NF-κB and glycemic markers concentrations in study groups

Groups	ND	ND + vitamin D	HFD	HFD + vitamin D	*P*-value
**Weight**	290 ± 30	256 ± 26.90	425 ± 3.71	381 ± 7.80	**0.001**
**TGF-β (ng/mg protein)**	1.1 ± 0.4	1.25 ± 0.4	1.11 ± 0.3	0.86 ± 0.34	**0.03**^**†**^
**MCP-1 (ng/mg protein)**	0.62 ± 0.15	0.53 ± 0.08	0.7 ± 0.07	0.38 ± 0.03	**0.002**^**†**^ **0.03**^**‡**^
**NF-kB**	0.01 ± 0.002	0.012 ± 0.002	0.012 ± 0.001	0.01 ± 0.001	0.04^*^ **0.03**^**†**^
**Glucose (mmol/l)**	7.3 ± 1.5	7.08 ± 3.2	10.37 ± 0.7	8.16 ± 2	0.11
**Insulin (mIU/l)**	9.4 ± 0.3	9.16 ± 1.04	13.2 ± 3.71	8.68 ± 1.43	0.04^*^ **0.01**^**†**^
**HOMA-IR**	0.63 ± 0.14	0.56 ± 0.16	1.25 ± 0.4	0.65 ± 0.22	0.006^*^ **0.008**^**†**^

Data are presented as means ± SD. Statistical differences between groups were assessed by one-way ANOVA followed by Tukey’s test for Post Hoc analysis; *TGF-β* transforming growth factor β; *MCP-1* monocyte chemoattractant protein-1; *NF-κB* nuklear factor kappa B. Bold values represent statistically significant threshold

† *P* value indicated inter group differences. *P*-value < 0.05 was considered as statistically significant

^†^ Significant differences between HFD + vitamin D and HFD

^*^ Significant differences between HFD and ND

^‡^ Significant difference between ND and ND + vitamin D group

**Table 2:** Correlation matrix between inflammatory factors of heart tissue and markers of glycemic status

Parameter	r (^†^P)	Glucose	Insulin	HOMA-IR
**NF-kβ**	r	0.18	0.18	0.078
	P	0.6	0.6	0.8
**TGF-β**	r	0.12	**0.63**	0.31
	P	0.7	**0.04**	0.3
**MCP-1**	r	-0.18	0.41	-0.012
	P	0.6	0.2	0.9

*TGF-β* Transforming growth factor β, *MCP-1* Monocyte chemoattractant protein 1, *NF-kβ* Nuclear factor kappa-β

^†^*P* values obtained by Pearson correlation analysis

*P* < 0.05 was considered as statistically significant

## Discussion

In the present study, five weeks administration of vitamin D had meaningful effects in reducing TGF-β, MCP-1 and NF-κB concentrations in the heart tissue of obese rats. Vitamin D decreased TGF-β concentrations in HFD + D group even more than ND group; this might be explained by vitamin D structure as a fat soluble vitamin which has a better absorption and metabolism in a high-fat diet. Although, HFD could not induce TGF-β elevation in comparison to ND, but the beneficial role of vitamin D in reducing TGF-β in HFD + vitamin D shouldn’t be ignored. Similar to our report, Sousa-Pinto, et al. characterized the patterns of TGF-β expression in fat depots of rats fed by a high fat diet in comparison to control group and evaluated the mRNA expression levels of TGF-β in all study groups by real-time polymerase chain reaction (PCR). They reported lower expression of TGF- β in retroperitoneal and epididymal adipose tissue in spite of feeding with high fat diet [37]. One possible explanation, is the differential effects of dietary carbohydrate versus dietary fat on pro-inflammatory markers; as we explained before, the composition of normal diet was 60% of carbohydrate, 30% of protein and 10% of dietary fat while high fat diet comprised of 59% of fat, 30% of carbohydrate and 11% of protein. In other published data from the current project, we demonstrated organ-specific effects of dietary carbohydrate and fat in producing inflammation in the human body; for example, high carbohydrate diet but not high fat diet, exerted strong effect in increasing TNF- α in cardiac tissue; whereas, the role of HFD in inducing pro-inflammatory response was more pronounced in renal tissues of rats [38] even though, in several human studies, dietary carbohydrate exerted more strong inflammatory response compared with dietary fat; in the study by Karimi E et al. [39] similar to our finding, high carbohydrate diet was positively associated with serum TGF-β and MCP-1 concentrations compared with dietary fat among 360 obese women while high fat diet was in negative association with TGF-β levels (β= -0.95, *P* = 0.05); possible role of carbohydrate in vascularization and angiogenesis might be attributed to the central role of it in TGF-β induction. Accordingly, the borderline non-significant increase in NF-κB activity in ND + vitamin D group compared with HFD group can be attributed to the higher carbohydrate amount of ND diet. This finding is in agreement of several previous studies that reported the potent positive role of dietary carbohydrate in increasing the receptor activator of the nuclear factor kappa-B ligand (RANKL) pathway [40, 41]. Moreover, vitamin D exerted reduced TGF-β concentrations in the cardiac tissue of HFD + vitamin D rats in our work. The role of TGF-β in myofibroblastic activation, increased collagen deposition and fibrosis in the myocardial injury has been reported previously [8, 9]. It has been suggested that gene therapy against TGF-β signaling pathways attenuates heart failure, left ventricular remodeling, and modulates infarcted tissue dynamics [8]. Vitamin D is an inhibitor of TGF-β expression, reduces cardiac myofibroblast activation, alters TGF-β pathway and reduces its bioavailability [10, 42, 43]. The anti-inflammatory role of vitamin D has also been shown previously [44, 45].

Vitamin D also demonstrated its anti-inflammatory properties against MCP-1 concentrations in HFD + vitamin D group. In agreement with our results, vitamin D caused a decline in the expression of MCP-1 in monocytes and down-regulated the lipopolysaccharide (LPS)-induced MCP-1 production in macrophages [46]. MCP-1 and its CCR2 receptor are considered as main targets of gene therapy for inflammation [47].

In the study by Mousa et al. [48] vitamin D had no effects on inflammatory parameters or NF-κB activity in peripheral blood mono-nuclear cells (PBMCs) of obese individuals. The possible reason for this controversy is the difference in the study procedure and design; the Moussa’s study was performed in vitamin D-deficient individuals and probably the dosage of vitamin D was insufficient to represent its anti-inflammatory actions; sixty-five overweight/obese, vitamin D-deficient (25-hydroxyvitamin D [25(OH)D] ≤ 50 nmol/L) adults were randomized to a single 100,000 IU bolus followed by 4,000 IU daily cholecalciferol or matching placebo for 16 weeks. Although in-vitro studies reported anti-inflammatory effects of vitamin D, this study didn’t show any effects of vitamin D supplementation on inflammatory markers or NFκB activity in-vivo in humans. However; more investigations are needed to reveal the underlying mechanisms of these controversies. Reduction of NF-κB after vitamin D supplementation in the current work could be explained by binding of vitamin D to VDR and suppressing NF-κB activation by direct interaction with IKKβ [49]. It has been shown that vitamin D reduces NF-kB translocation to the nucleus and reduces smooth muscle cell proliferation [50]. In the current study, vitamin D also improved markers of glycemic status. In our previous work, vitamin D represented amelioration in lipid abnormalities [35].

## Conclusion

This work for the first time, revealed the ameliorative effects of vitamin D against inflammation in the cardiac tissue of obese rats. Further investigation in human models are warranted to confirm the findings of the current study.

### Limitations

The results of the current study could not be generalized to human. Therefore, similar studies in human models are warranted. Moreover, it is suggested to evaluate histologic changes in cardiac tissue of rats for better clarification of underlying mechanisms.

## Data Availability

The data that support the findings of this study are available from corresponding author but restrictions apply to the availability of these data, which were used under license for the current study, and so are not publicly available. Data are however available from the authors upon reasonable request and with permission of corresponding author.

## Abbreviations

ANOVA: Analysis of variance
CAD: Coronary artery disease
CVD: Cardiovascular disease
HDL: High density lipoprotein cholesterol
LDL: Low density lipoprotein cholesterol
MDA: Malondialdehyde
NADH: Nicotinamide adenine dinucleotide
TC: Total cholesterol
TGF-β: Transforming growth factor β
TNF-α: Tumor necrosis factor α
MCP-1: Monocyte chemoattractant protein 1
IL-6: Interleukin 6
